# Reversible Cardio‐Renal‐Cerebral Syndrome in a Dog: A Case Report

**DOI:** 10.1111/jvim.70249

**Published:** 2025-10-01

**Authors:** Gianira Candelario, George Kramer, Brienne Williams, Samantha Seies, Nikki Gaudette, Laura Patterson Rosa

**Affiliations:** ^1^ Department of Veterinary Clinical Sciences, Lewyt College of Veterinary Medicine Long Island University Brooklyn New York USA; ^2^ Atlantic Coast New York Veterinary Specialists Bohemia New York USA

**Keywords:** bradycardia, canine, hyperkalemia, third‐degree AV block

## Abstract

A 14‐year‐old miniature Pinscher was presented with azotemia, severe hyperkalemia, and oliguria caused by decompensation of chronic renal disease, along with bradycardia resulting from third‐degree atrioventricular (AV) block. Supportive medical treatment was instituted for the oliguria, azotemia, and hyperkalemia. Within 12 h of hospitalization, multifocal central neurologic signs developed, including nystagmus, quadriparesis, decreased mentation, and ventral neck flexion. A diagnosis of cardiorenal cerebral syndrome was made. A transvenous permanent pacemaker was placed to improve cardiac output. Within 24 h, clinical signs improved, and the patient was discharged from the hospital on day three.

**Abbreviations**
ACNYVS: Atlantic Coast New York Veterinary Specialists
AKI: acute kidney injury
AV: atrioventricular
BUN: blood urea nitrogen
CKD: chronic kidney disease
ECG: electrocardiogram
LA: left atrial
LV: left ventricular
O: oliguric
RA: right atrial
RRT: (requiring) renal replacement therapy
SQ: subcutaneous
USG: urine specific gravity

## 1 Introduction

Cardiorenal syndrome (CRS) or cardiovascular‐renal disorders (CvRD) are characterized by impairment of the kidneys or heart or both, resulting in dysregulation of physiological interactions and contributing to progressive dysfunction in one or both organs [1, 2]. This bidirectional relationship has been studied extensively in human medicine, leading to a classification system with five subtypes based on primary and secondary organ involvement, [3] and a consensus statement in veterinary medicine delineating three subtypes: renal dysfunction secondary to primary cardiovascular disease (CvRD_H_), cardiovascular dysfunction resulting from primary renal disease (CvRD_K_), and concurrent impairment of both systems due to primary disease affecting both the cardiovascular and renal systems (CvRD_O_) or as a consequence of external factors such as drugs, toxins, or toxicants [2]. Although the heart‐kidney axis role in CRS is well documented, the interplay among the cardiac, renal, and cerebral systems, termed *cardiorenal cerebral syndrome*, is less well understood. In humans, acute or chronic dysfunction in one of these organs can adversely affect the others, creating a complex clinical scenario. For example, acute heart failure can lead to renal impairment and cerebral hypoperfusion, exacerbating patient morbidity and mortality [4].

In veterinary medicine, particularly in dogs, literature on CRS and its cerebral implications is sparse, despite the noted prevalence of CvRD in some breeds such as the miniature Poodle (9.32%), Yorkshire Terrier (8.5%), Shih‐Tzu (4.24%), and Pinscher (4.24%) [5]. In humans, cardiorenal cerebral syndrome has been described in a patient secondary to an acute decrease in cardiac output from complete atrioventricular (AV) block leading to renal and cerebral hypoperfusion [6] and in two cases of oliguric acute kidney injury (AKI) secondary to third‐degree AV block that resolved after pacemaker implantation [7, 8]. Evaluation of cerebral‐cardiac syndrome in canine models of traumatic brain injury also demonstrates the crucial role of addressing cardiac dysfunction to treat acute neurologic damage [9]. However, comprehensive details regarding treatments addressing the primary role of cardiac, renal, or nervous systems in dogs are notably lacking [2]. Despite the likely rarity of this condition, recognizing and understanding the mechanisms underlying cardiorenal cerebral syndrome in dogs is crucial for adequate therapeutic strategies.

Our case report aims to bridge the existing knowledge gap by presenting a case of reversible cardiorenal cerebral syndrome in a dog. We describe the pathophysiological interactions among the heart, kidneys, and brain, contributing to a broader understanding and possible treatment of this complex syndrome in veterinary medicine.

## 2 Patient Information

A 14‐year‐old, 4.3 kg, male intact Miniature Pinscher was referred to the Emergency and Critical Care service of Atlantic Coast New York Veterinary Specialists (ACNYVS) with a 2‐day history of lethargy, inappetence, diarrhea, anorexia, and decreased urination of < 12 h in duration. The dog had been treated 2 weeks before for chronic active pancreatitis and was reported to be mildly azotemic with a serum creatinine concentration of 1.7 mg/dL (normal, 0.5–1.6 mg/dL) and BUN concentration of 108 mg/dL (normal, 6–31 mg/dL). The referring veterinarian also noted marked bradycardia of 20 beats/min (normal, 70–120 bpm) [10]. No prior cardiac abnormalities had been documented, and although a history of chronic renal disease was noted by the referring veterinarian, the precise onset was undetermined, and corresponding renal function test results were not provided. Additionally, the progression and therapeutic management of the renal disease was not documented.

## 3 Clinical Findings

Physical examination upon presentation to ACNYVS identified mild dehydration (3%–5%), hypothermia 34.3°C (normal, 37.5°C–39.2°C), marked resting bradycardia (20 bpm, normal, 70–120 bpm), mild tachypnea of 30 breaths/min (normal, 18–34 breaths/min), pale mucous membranes, and a grade II/VI systolic heart murmur. An ECG using a Dre/True ECG 12T HP Hewlett Packard M1705A disclosed third‐degree AV block, non‐responsive to an atropine response test using 0.04 mg/kg IM [11]. Thoracic radiographs indicated a vertebral heart score of 11, within the normal reference interval for the breed (9.6–12.2) [12] and a mild bronchial pattern. Blood samples were collected for CBC and serum biochemistry, as well as urine samples by catheterization for clinical pathology evaluation (Bloodhound Laboratories, Bohemia, NY). The CBC showed mild neutrophilia 13.1 K/μL (normal, 2.9–12.0 K/μL) and lymphopenia 0.4 K/μL (normal, 0.8–5.1 K/μ/L; Table 1). Serum biochemistry identified markedly increased serum creatinine concentration 3.4 mg/dL (normal, 0.5–1.7 mg/dL), indicating renal impairment along with hyperphosphatemia 10.6 mg/dL (normal, 2.9–5.3 mg/dL), severe hyperkalemia 9.7 mEq/L (normal, 3.9–5.1 mEq/L), hyperglycemia 230 mg/dL (normal, 76–119 mg/dL), mild hyponatremia 137 mEq/L (normal, 142–152 mEq/L), and metabolic acidosis. Urinalysis indicated isosthenuria 1.017 (normal, 1.016–1.060) with proteinuria +3 (normal, negative) and calcium oxalate monohydrate crystals 4–10/hpf (high power field; normal, 0–5/hpf), indicating impaired renal function (Table 1) [10].

**TABLE 1:** Blood, serum, and urinalysis results and respective reference intervals patient admission at the ACNYVS.

Variable	Patient result	Reference interval [10]
Neutrophils (segmented WBC)	13.1 K/μL	2.9–12.0 K/μL
Lymphocytes	0.4 K/μL	0.8–5.1 K/μL
Platelets	442 K/μL	186–545 K/μL
Blood urea nitrogen (BUN)	122 mg/dL	8–28 mg/dL
Creatinine	3.4 mg/dL	0.5–1.7 mg/dL
Phosphorus	10.6 mg/dL	2.9–5.3 mg/dL
Potassium	9.7 mEq/L	3.9–5.1 mEq/L
Glucose	230 mg/dL	76–119 mg/dL
Sodium	137 mEq/L	142–152 mEq/L
Bicarbonate	13 mEq/L	17–24 mEq/L
Urine specific gravity (USG)	1.017	1.016–1.060
Urine protein	+3	Negative
Calcium oxalate monohydrate crystals	4–10/hpf	0–5/hpf

The dog was hospitalized overnight and monitored via ECG telemetry (Dre/True ECG 12T HP Hewlett Packard M1705A). Supportive care included 40%–50% supplemental oxygen and IV fluids consisting of 0.45% NaCl with 2.5% dextrose administered at 60 mL/kg/day. This fluid regimen was selected to address existing hyponatremia while minimizing the risk of extracellular fluid volume overload. Antimicrobial treatment included ampicillin/sulbactam at 22 mg/kg IV q8h (UNASYN, Pfizer Inc., New York, NY) and metronidazole at 10 mg/kg IV q12h.

Urine output was monitored using indirect methods, including evaluating serial body weight, assessing urination frequency and volume, recording fluid intake, and observing for clinical signs of dehydration. No urination was noted overnight after admission, and findings were consistent with International Renal Interest Society (IRIS) AKI grade III, subgrade oliguric state (defined as < 0.3 mL/kg/h for > 24 h or anuria for > 12 h). An indwelling urinary catheter was recommended to accurately monitor fluid balance, but the owner declined this intervention. As a result, urine output was estimated based on visual observation and caregiver reports. During the overnight period, the patient also deteriorated neurologically with severely decreased mentation, vertical nystagmus, ventral dorsoflexion of the neck, and non‐ambulatory status.

The morning after initial hospitalization, the dog was transferred to the internal medicine service, and a request for cardiology and neurology consultations was made because of the onset of neurologic signs noted by the attending emergency and critical care veterinarian. A targeted abdominal ultrasound examination of the urinary bladder indicated decreased size based on expected volume for the species (patient, < 0.5 mL/kg; normal, ≥ 0.5 mL/kg) [13]. The internal medicine veterinarian noted persistent dehydration along with dry mucous membranes, hypothermia (35.6°C), mildly increased respiratory effort, and the previously noted severe bradycardia. A grade II/VI systolic murmur with the point of maximum intensity at the left fifth intercostal space also was noted, suggesting mitral regurgitation. The neurologic clinical signs also were confirmed.

## 4 Diagnostic Assessment

Because of the patient's clinical presentation and progressive clinical signs, additional diagnostic tests were pursued, including an echocardiogram (Philips EPIQ CVx3D, Philips, Amsterdam, Netherlands) and an abdominal ultrasound examination (Versana Balance, GE HealthCare, Chicago, IL). Echocardiogram findings are further discussed in the supplemental file. Assessment indicated myxomatous mitral valve disease (MMVD) classified as Stage B2 following the American College of Veterinary Internal Medicine (ACVIM) guidelines.

Echocardiographic evidence of substantial cardiac enlargement as a result of volume overload, moderate pulmonary hypertension, third degree AV block, and stage B2 MMVD was noted, suggesting that chronic mitral regurgitation and volume overload had led to chamber dilatation and pulmonary hypertension. Abdominal ultrasound examination indicated moderately decreased renal corticomedullary distinction bilaterally without any hydronephrosis, renoliths, renal cysts, or masses, and was suspected to be secondary to acute on chronic kidney disease (CKD). A 3 cm irregular, heterogeneous, cavitated left adrenal gland mass was noted with no invasion into the caudal vena cava. The differential diagnoses for this mass included adrenocortical tumor or pheochromocytoma. The right adrenal gland appeared normal. Further evaluation for the suspected left adrenal mass was recommended but declined by the owner. The pancreas was mottled and featured hyperechoic nodular changes in the right lobe consistent with chronic pancreatitis [14].

Because of hyperkalemia, azotemia, and oliguria in the presence of persistent dehydration, IV fluids were increased to 85 mL/kg/day, and 0.3 U/kg insulin‐R (Humulin, Lilly USA LLC, Indianapolis, IN) was administered with 1.4 mL/kg of 50% dextrose solution. A repeat biochemistry profile was performed 1 h after insulin administration and indicated worsening azotemia (BUN concentration, 151 mg/dL; serum creatinine concentration, 4.0 mg/dL compared with admission results of 122 and 3.4 mg/dL, respectively), progressive hyperphosphatemia (15.8 mg/dL as compared with 10.6 mg/dL at admission), and slight improvement in severe hyperkalemia (8.5 mEq/L; admission, 9.7 mEq/L). The acute onset of renal failure was classified as IRIS AKI grade III (oliguria, proteinuria) on suspected preexisting CKD [15]. Baseline systolic blood pressure was not documented.

**FIGURE 1:**
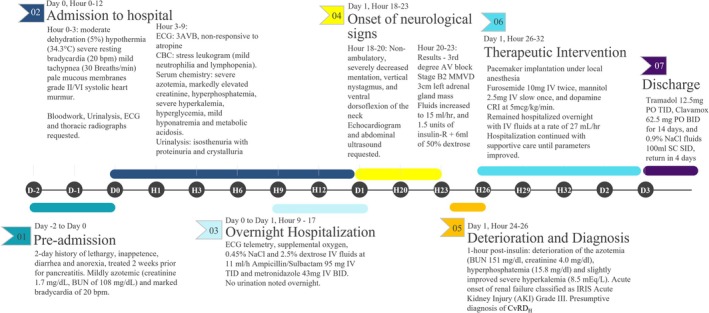
Case timeline. Days are represented by “D,” and hours within the described days are represented by “H.”

Given the case presentation, a presumptive diagnosis of CvRD_H_ was made. According to the neurologic assessment, the multifocal central neurologic signs were likely caused by metabolic derangements associated with renal failure and hypoperfusion of the brain because of decreased cardiac output. Based on these findings, the diagnosis was amended to cardiorenal cerebral syndrome [6] (see Figure 1 for clinical timeline).

## 5 Therapeutic Intervention

Based on preliminary outcomes in humans, the decision was made to proceed with permanent pacemaker implantation under local anesthesia with the hypothesis of CvRD_H_, and to decrease the risk of decreasing blood pressure and perfusion and further exacerbating the renal failure, hyperkalemia, and neurologic signs. A permanent Medtronic Adapta pacemaker, model CentralVue CV10 (Medtronic Inc., Minneapolis, MN), preferred as the definitive treatment for third‐degree AV block, was successfully implanted under local anesthesia using a local lidocaine block and low‐dose ketamine and valium, with pacemaker beats noted and a heart rate of 80 bpm. Post‐operatively, the dog remained quiet, alert, and responsive, and both ventral dorsoflexion and nystagmus resolved. Systolic blood pressure was measured at 120 mmHg using an ultrasonic Doppler flow detector model 811‐B (Parks Medical, Electronics Inc., Aloha, OR).

At this time, the dog remained oliguric despite stabilized hydration and an increase in body weight to 4.5 kg. The dog received furosemide at 2.2 mg/kg IV twice (the dog was normally hydrated), mannitol 0.55 mg/kg IV slowly once, and was started on a dopamine continuous rate infusion (CRI) at 5 mcg/kg/min and remained hospitalized overnight, with IV fluids administered at a rate of 120 mL/kg/day. The goal was to stimulate urine production and convert oliguric AKI into a non‐oliguric state. In addition, the dose of ampicillin/sulbactam was decreased to 22 mg/kg IV q12h (UNASYN, Pfizer Inc., New York, NY) because of decreased kidney function, and metronidazole was administered at 10 mg/kg IV q12h. Physical examination was conducted the following morning, and the patient showed improved mentation and appeared more alert, but still was non‐ambulatory, with rightward postural instability when attempting to stand. A follow‐up serum biochemistry profile indicated improving hyperkalemia (6.7 mEq/L), azotemia (BUN concentration, 129 mg/dL; serum creatinine concentration, 3.4 mg/dL), and hyperphosphatemia (14.6 mg/dL). Urine output returned to normal, and the dopamine CRI was tapered and discontinued. The dog remained hospitalized overnight, receiving IV fluids, hydromorphone IV, and ampicillin/sulbactam, and metronidazole PO. During hospitalization, mentation continued to improve, and the dog became ambulatory with assistance.

The dog was discharged on the third day with tramadol hydrochloride 2.8 mg/kg PO q8h or as needed for pain, amoxicillin trihydrate/clavulanate potassium (CLAVAMOX, Beecham Inc., Bristol, TN) 13.75 mg/kg PO q12h for 14 days, and 0.9% NaCl 100 mL SC q24hr because of persistent but improved azotemia. Findings during reevaluations performed 4 and 11 days after discharge are presented in the supplemental file. On physical examination reevaluation 18 days after initial discharge, the dog had normal mentation, was ambulatory with no ataxia, had a heart rate of 80 bpm, with grade III/VI systolic heart murmur with point of maximal intensity at the left fifth intercostal space, and was eupneic with normal auscultation. A renal profile indicated mild azotemia (BUN concentration, 56 mg/dL) and slightly increased serum creatinine concentration (1.8 mg/dL) as well as slightly improved hyperkalemia (6.4 mEq/L). The owner was instructed to finish treatment with SC fluids and to return the dog for reevaluation in 3 months.

## 6 Discussion

Cardiorenal syndrome has been well described in humans, and subdivided into five types (further described in Data S1) [1]. In dogs and cats, CvRD have been described and classified in a consensus statement [2, 16]. Considering the case presentation, outcome, and disease characterization, the dog of our case report falls within the classifications of CvRD_H_ and CRS Type 1.

**FIGURE 2:**
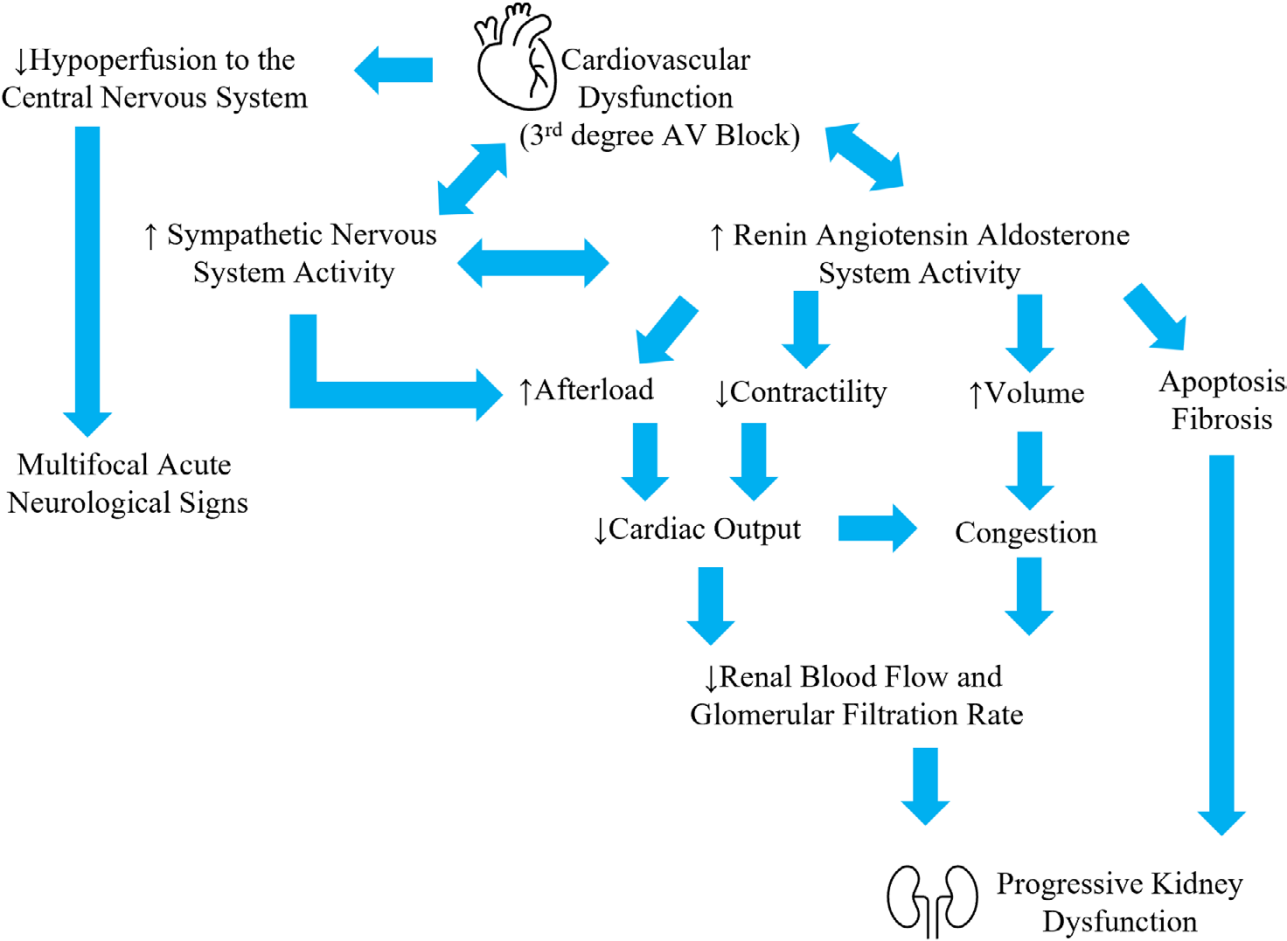
Postulated mechanisms underlying the CvRD_H_ relationship between the heart and kidneys, based on the suggested mechanisms from the consensus statement [2].

For this dog, the severe bradycardia was likely secondary to the third‐degree AV block, which may have led to hyperkalemia from decreased renal perfusion (Figure 2). Hyperkalemia can result from a wide variety of conditions that either cause a shift of potassium ions from intracellular to extracellular fluid or a decrease in the renal excretion of potassium, and the majority of dogs (55.6%) that have serum sodium:potassium (Na:K) ratios < 20 are in renal failure [17]. Although potassium homeostasis is determined by dietary intake and renal excretion of potassium [18], hyperkalemia in renal insufficiency or conditions with renal hypoperfusion can be attributed to decreased ability to excrete the potassium load [19, 20]. Hyperkalemia can lead to arrhythmias and conduction disturbances such as sinus bradycardia, sinus arrest, sino‐ventricular rhythm, AV block, and asystole [21]. Still, an acute decrease in cardiac output may result in a decreased glomerular filtration rate (GFR), increased serum creatinine and BUN concentrations, and decreased urine output [22] similar to the clinical and pathological presentation in the dog described here.

Cardiac sensitivity to hyperkalemia is maximal in the sinus node and atrial myocardium in dogs [23, 24]. As serum potassium concentration increased, the P‐wave becomes flattened and eventually lost, followed by prolongation of the PR interval and QRS duration [25]. In our case, even after hyperkalemia slightly improved following insulin and fluid therapy, the dog continued to exhibit complete third‐degree AV block, further supporting the premise that the cardiovascular dysfunction was primary and not the result of hyperkalemia. Permanent pacemaker implantation resulted in increased heart rate, improved cardiac output, and renal perfusion, resolution of neurologic signs, and partial improvement of hyperkalemia, which persisted until the final reevaluation. These findings suggest that renal and cerebral injury may be reversible after pacemaker placement [6]. A single case of severe bradyarrhythmia‐induced hyperkalemia and increased BUN was reported in a dog with third‐degree AV block and also resolved after pacemaker implantation [21]. In conclusion, we have described a case of cardiorenal cerebral syndrome secondary to decreased cardiac output associated with bradycardia resulting from third‐degree AV block, leading to hypoperfusion of the kidneys and nervous system and triggering multi‐systemic pathology. Because the observed pathology originates primarily from cardiac dysfunction, it can be reversed by correcting the bradycardia. Improved recognition of the pathologic origin can expedite appropriate medical treatment, leading to better patient outcome.

## Disclosure

Authors declare no off‐label use of antimicrobials.

## Ethics Statement

Authors declare no institutional animal care and use committee or other approval was needed. Authors declare human ethics approval was not needed.

## Conflicts of Interest

The authors declare no conflicts of interest.

## Supporting information


**Data S1:** Supporting Information.
