# Correction to “Reversible Cardio‐Renal‐Cerebral Syndrome in a Dog: A Case Report”

**DOI:** 10.1111/jvim.70263

**Published:** 2025-10-28

G. Candelario, G. Kramer, B. Williams, S. Siess, N. Gaudette, and L. Patterson Rosa, “Reversible Cardio‐Renal‐Cerebral Syndrome in a Dog: A Case Report,” *Journal of Veterinary Internal Medicine* 39 (2025): e70249, https://doi.org/10.1111/jvim.70249.

In the above mentioned article, author name Samantha Seies has been corrected to Samantha Siess.

The original article has also been corrected to reflect this change.

We apologize for this error.

