# Supplementary material for: Reversible Cardio‐Renal‐Cerebral Syndrome in a Dog: A Case Report
Source: J Vet Intern Med. 2025 Oct 1;39(6):e70249. doi: 10.1111/jvim.70249 (PMC12489176; doi:10.1111/jvim.70249)
Supplement: Supplementary file 1 — Data S1: Supporting Information. [file JVIM-39-e70249-s001.docx]

**Echocardiogram Findings**

The echocardiogram showed: Echo measurements: Ao: 0.9 cm, LA: 2.0 cm, LA/Ao 2.22, LVID: 2.1 cm (with apical sphericalization), LVIDdN: 1.7 cm, LVIDs:1.1 cm, IVSd: 0.6 cm, LVPWd: 0.55 cm, FS: 50%, Ao Vel: 1.1 m/s, E/A: 1.38, E Vel: 1.1 m/s, A Vel: 0.8 m/s, TR Vel: 4.0 m/s. Marked left atrial (LA) dilation, moderate left ventricular (LV) dilation, moderate right atrial (RA) dilation and a mild right ventricular (RV) dilation, despite an adequate contractility based on published reference intervals [14]. The mitral valve and the tricuspid valves were mildly thickened [15] and there were moderate mitral and tricuspid regurgitations present. Estimated systolic pulmonary pressure was calculated to be 64mmHg (moderate pulmonary hypertension). This is based on the TR pressure gradient calculated using the modified Bernoulli equation. The IVSd and the LVPWd both indicate mild hypertrophy that could be compensatory hypertrophy or in response to increased afterload. The normalized LVIDd (LVIDdN) was calculated using the formula LVIDdN= LIVDd (cm)/Body weight (kg)^0.332^ recommended for use in small breed dogs <5kg. The combination of mitral regurgitation, pulmonary hypertension, and third degree AV block contributes to reduced cardiac output, systemic venous congestion, and renal dysfunction [2].

**Recheck Findings**

Recheck examination was scheduled for 4 days after discharge, where physical examination revealed the patient was BAR with normal mentation, ambulatory with mild residual ataxia, temperature of 100.4°F, heart rate of 80 bpm, grade III/VI systolic heart murmur (SHM) with the point of maximal impulse (PMI) at the left 5th intercostal space (ICS), and lungs were clear and eupneic. Additional renal panel revealed improved azotemia (BUN 75 mg/dL, creatinine 2.8 mg/dL), mild hyperphosphatemia (6.9 mg/dL), mild hyperkalemia (5.7 mEq/L). Continued treatment with Amoxicillin trihydrate/clavulanate potassium (CLAVAMOX®, Beecham, Inc., Bristol, TN) and .9% NaCl fluids 100ml SC was recommended, and a recheck scheduled for 7 days.

During the recheck 11 days after the initial discharge, the physical examination revealed BAR mentation, ambulatory with minimal ataxia present, heart rate of 80 bpm, grade III/IV SHM PMI left 5th ICS and lungs were clear and eupneic. A renal panel revealed slightly improved azotemia (BUN 74 mg/dL, creatinine 2.3 mg/dL) and deteriorated hyperkalemia (6.8 mEq/L). Pacemaker interrogation revealed 92.8% paced and 7.2% sensed. The patient was dismissed with recommended treatment with SQ fluids continued and the return in one week for one more recheck exam and supplementary renal panel.

**Supplemental Discussion**

CRS has been well described in humans, and subdivided into five types: Type 1, acute impairment of heart function resulting in decreased cardiac output leading to acute kidney injury (AKI); Type 2, chronic disease of the heart leading to kidney injury, such as seen in patients with chronic heart failure; Type 3, acute worsening of kidney function resulting in impaired cardiac function or injury; Type 4, chronic kidney disease (CKD) resulting in cardiac injury; and Type 5, in which systemic disease condition(s) results in simultaneous damage to both the heart and kidney [1].

The significance of the adrenal tumor in this case is unclear. The differential diagnoses included adrenocortical tumor versus a pheochromocytoma. Despite recommendations of adrenal testing, plasma-free metanephrine/normetanephrine, urine catecholamine:creatinine ratio and ultrasound-guided fine needle aspiration or biopsy of the mass followed by possible surgical resection, the owners declined workup of the adrenal tumor. Thus, it is possible that if the tumor was a pheochromocytoma it could have been associated with the observed arrhythmia [30-32], yet the exact mechanism as to how it affects the cardiovascular system remains unclear.

In humans, while the cardiac and renal components of cardiorenal cerebral syndrome have been extensively explored, the cerebral aspect remains underrepresented. Cardiorenal cerebral syndrome involves a complex interplay of neurohumoral activation, autonomic dysfunction, systemic inflammation, fluid/electrolyte imbalances, and hypothalamic alterations. Neurohumoral responses are characterized by heightened activity of the sympathetic nervous system and the renin-angiotensin-aldosterone system (RAAS), leading to increased sympathetic outflow that contributes to hypertension and imposes additional stress on cardiac and renal systems. Concurrently, impairment in autonomic reflexes disrupts heart rate variability and blood pressure regulation, further exacerbating cardiac and renal dysfunction [33].

Inflammatory cytokines released from cardiac and renal dysfunction can impact the brain, causing neuroinflammation, while the diminished cardiac output leads to reduced cerebral perfusion, compromising oxygen and nutrient delivery. These predominantly affect the frontal cortex and hippocampus, causing white matter lesions, cortical atrophy and triggering cognitive deficits ranging from mild impairment to overt dementia [34]. In addition to hypoperfusion, hyperkalemia may further aggravate neurological dysfunction, affecting neuronal membrane potentials, leading to confusion, lethargy, muscle weakness, and in severe cases, seizures [35]. Hyperkalemia can be a silent yet dangerous contributor to neuromuscular and neurological impairment, particularly in patients with compromised renal and cardiac function [36].

Furthermore, bradycardia, while commonly associated with vasovagal syncope due to transient cerebral hypoperfusion, has also been implicated in more insidious forms of neurological deterioration in the absence of hypotension or syncopal episodes. Sustained reductions in heart rate may contribute to chronic cerebral underperfusion and subsequent cognitive decline. Notably, elderly patients with severe bradycardia demonstrated significant improvements in mental status following pacemaker implantation, suggesting that cardiac rhythm stabilization can improve cognitive symptoms [37] and reiterating the critical role of cardiac pacing in mitigating neurocognitive impairment in patients with cardiac-origin cerebral dysfunction.

References

1 Rangaswami J, Bhalla V, Blair JEA*, et al.* Cardiorenal Syndrome: Classification, Pathophysiology, Diagnosis, and Treatment Strategies: A Scientific Statement From the American Heart Association. *Circulation* 2019; **139:** e840-e78.

16 Rosenstein DS. Diagnostic imaging in canine pheochromocytoma. *Veterinary radiology & ultrasound* 2000; **41:** 499-506.

29 Barthez PY, Marks SL, Woo J, Feldman EC, Matteucci M. Pheochromocytoma in dogs: 61 cases (1984–1995). *Journal of Veterinary Internal Medicine* 1997; **11:** 272-8.

30 Zweiker R, Tiemann M, Eber B*, et al.* Bradydysrhythmia‐related presyncope secondary to pheochromocytoma. *Journal of internal medicine* 1997; **242:** 249-53.

31 Edmondson EF, Bright JM, Halsey CH, Ehrhart EJ. Pathologic and cardiovascular characterization of pheochromocytoma-associated cardiomyopathy in dogs. *Veterinary pathology* 2015; **52:** 338-43.

32 Mak G, Allen J. Simultaneous pheochromocytoma and third-degree atrioventricular block in 2 dogs. *Journal of Veterinary Emergency & Critical Care* 2013; **23**.

33 Patel KP, Katsurada K, Zheng H. Cardiorenal Syndrome: The Role of Neural Connections Between the Heart and the Kidneys. *Circ Res* 2022; **130:** 1601-17.

34 Mene-Afejuku TO, Pernia M, Ibebuogu UN, Chaudhari S, Mushiyev S, Visco F, Pekler G. Heart Failure and Cognitive Impairment: Clinical Relevance and Therapeutic Considerations. *Curr Cardiol Rev* 2019; **15:** 291-303.

35 Frey A, Sell R, Homola GA*, et al.* Cognitive deficits and related brain lesions in patients with chronic heart failure. *JACC: Heart Failure* 2018; **6:** 583-92.

36 Weiner ID, Wingo CS. Hyperkalemia: a potential silent killer. *J Am Soc Nephrol* 1998; **9:** 1535-43.

37 Koide H, Kobayashi S, Kitani M, Tsunematsu T, Nakazawa Y. Improvement of cerebral blood flow and cognitive function following pacemaker implantation in patients with bradycardia. *Gerontology* 1994; **40:** 279-85.
